# Comprehensive geriatric assessment measures and subsequent EMS-transported emergency department use in adults aged ≥ 80 years: a retrospective cohort study

**DOI:** 10.1186/s12873-026-01590-z

**Published:** 2026-04-18

**Authors:** Ahmet Aykut, Cüneyt Güven, Ertuğ Günsoy, Cem Yıldırım, Mehmet Şirin Büyükkaya, Mehmet Yorgun, Osman Taş, Mahmut Şahin

**Affiliations:** 1 Department of Emergency Medicine, SBU Van Education and Research Hospital, Van, Türkiye; 2 Department of YAŞAM, SBU Van Education and Research Hospital, Van, Türkiye

**Keywords:** Frailty, Geriatric assessment, Emergency Medical Services, Emergency service hospital, Aged 80 and over

## Abstract

**Background:**

Emergency Medical Services (EMS) ambulance transport is a common route of emergency department (ED) arrival for adults aged ≥ 80 years, but factors associated with future EMS-transported ED presentation remain uncertain. We assessed whether baseline comprehensive geriatric assessment (CGA) measures were associated with EMS-transported ED presentation within 12 months.

**Methods:**

We performed a retrospective cohort study in Türkiye linking the Healthy Aging Center (YAŞAM) CGA registry, which records routine multidomain geriatric assessments in community-dwelling older adults, to ED administrative records from a tertiary hospital. Consecutive adults aged ≥ 80 years who underwent baseline CGA between October 5, 2023 and December 10, 2024 were included (*n* = 587). The primary outcome was ≥ 1 EMS-transported ED presentation within 12 months; repeat use (≥ 2 presentations) was a prespecified secondary outcome. Separate age- and sex-adjusted domain-specific association logistic regression models were fitted for each CGA measure; exploratory age- and sex-adjusted logistic models were also used for repeat use. Discrimination was assessed using the area under the receiver operating characteristic curve (AUC). Cause-specific Cox models were used as sensitivity analyses for time to first EMS-transported ED presentation, censoring at death before any EMS event.

**Results:**

During follow-up, 159/587 participants (27.1%) had ≥ 1 EMS-transported ED presentation, 55/587 (9.4%) had repeat use, and 51/587 (8.7%) died. Higher frailty was associated with the primary outcome (odds ratio [OR] per 1-point increase, 1.30; 95% confidence interval [CI], 1.16–1.47), whereas higher Katz Activities of Daily Living scores (OR, 0.77; 95% CI, 0.70–0.85), Mini Nutritional Assessment-Short Form scores (OR, 0.85; 95% CI, 0.80–0.92), and Timed Up and Go indicator = 1 (OR, 0.34; 95% CI, 0.23–0.50) were associated with lower odds. Discrimination was modest across models (AUC, 0.575–0.643). Cause-specific Cox sensitivity analyses showed directionally similar associations.

**Conclusions:**

In adults aged ≥ 80 years, multiple routinely collected CGA domains were associated with subsequent EMS-transported ED use. These measures should be interpreted as indicators of geriatric vulnerability rather than stand-alone predictive tools; any predictive application would require externally validated multivariable models.

**Supplementary Information:**

The online version contains supplementary material available at 10.1186/s12873-026-01590-z.

## Introduction

Population ageing is increasing the demand for urgent and unscheduled care, and many emergency department (ED) encounters in older adults begin with Emergency Medical Services (EMS) activation. Older adults account for a substantial and growing share of ambulance contacts, and falls alone generate considerable EMS workload in ageing populations [1, 2]. In addition, a relatively small subgroup of frequent EMS users can account for a disproportionate share of activations, making the identification of clinically vulnerable older adults relevant not only for patient care but also for service planning [3].

A plausible explanation for this pattern is that EMS use in very old adults is shaped not only by acute diagnoses, but also by underlying multidomain geriatric vulnerability. Frailty reflects reduced physiological reserve and increased vulnerability to stressors, helping to explain why older adults with apparently similar illnesses may have markedly different trajectories of decompensation, recovery, and service needs [4–6]. In parallel, impairments in function, mobility, cognition, mood, and nutrition may reduce the capacity for self-management, delay timely help-seeking, increase fall susceptibility, and lower the practical threshold for ambulance transport when acute symptoms occur [7–9]. Prehospital and ED studies have shown that frailty can be measured in emergency settings and is associated with clinically relevant outcomes, supporting the broader concept that vulnerability phenotyping may add information beyond chronological age alone [4, 7, 10].

However, an important evidence gap remains. Existing literature has largely focused either on frailty assessment in ambulance or ED settings, or on selected emergency outcomes, rather than on whether routinely collected multidomain comprehensive geriatric assessment (CGA) data obtained before an emergency episode are associated with subsequent EMS utilization in the “oldest-old” population [4, 7, 10]. The institutional Healthy Aging Center (YAŞAM) registry provides routine CGA data across frailty, basic and instrumental function, cognition, mood, nutrition, and mobility/performance in community-dwelling adults aged ≥ 80 years, creating an opportunity to examine whether baseline geriatric vulnerability is associated with later EMS-transported ED use under real-world care conditions [11]. We selected EMS-transported ED presentation, rather than ED presentation more broadly, as the primary outcome because ambulance transport represents a clinically and operationally distinct pathway of emergency care use in very old adults. In this population, EMS involvement may better capture vulnerability-related emergency reliance linked to impaired mobility, functional dependence, and reduced self-transport capacity. It is also particularly relevant to service planning because EMS use represents a specific component of emergency care demand. We acknowledge that this approach does not capture older adults who reach the ED through non-EMS pathways. Therefore, we aimed to evaluate whether baseline CGA measures recorded in adults aged ≥ 80 years were associated with subsequent EMS-transported ED presentation over 12 months. We also aimed to describe repeat EMS use patterns and to summarize administratively coded reasons for presentation descriptively, without treating them as adjustment variables on the causal pathway between baseline geriatric status and EMS utilization.

## Methods

### Study design and setting

We conducted a single-center retrospective cohort study at a tertiary academic hospital in Türkiye. The source population was assembled from the institutional YAŞAM registry, in which CGAs are routinely recorded as part of clinical care. The registry analyzed in this study was the institutional database of our hospital’s Healthy Aging Center and should not be interpreted as a regional or national dataset. Accordingly, the analytic cohort reflects a single-site care context in one tertiary hospital setting. Of 671 individuals registered in YAŞAM, 605 had an eligible registry entry within the prespecified baseline period (October 05, 2023 to December 10, 2024). Eighteen of these were excluded because no CGA had been performed, yielding a final analytic cohort of 587 participants. A study flow diagram is provided in Supplementary Figure S1. We defined the index date (t0) as the date of the baseline YAŞAM assessment recorded during the study period. Outcomes were ascertained within a fixed 12-month follow-up window after t0 using the hospital information system, and the latest follow-up endpoint occurred on December 10, 2025. We reported the study in accordance with the STROBE guideline for observational studies.

### Participants/subjects

We included consecutive adults aged ≥ 80 years with a documented baseline YAŞAM CGA during the prespecified study period. Cohort inclusion was based on eligibility within the YAŞAM registry rather than on subsequent ED use. No eligible records were excluded because of duplicate baseline assessments or invalid institutional identifiers; all included participants could be linked to the institutional ED records. We retained individuals with documented death status in institutional records and did not exclude them from the cohort, because outcomes were defined within a prespecified 12-month observation window.

### YAŞAM registry and assessment procedures

YAŞAM is an institutional Healthy Aging Center that provides routine multidomain geriatric assessment and follow-up for adults aged ≥ 80 years. Referrals occur through self-presentation, outpatient/polyclinic referrals, and home healthcare services according to local clinical practice, with the aim of enabling safe longitudinal follow-up and, when appropriate, assessment without requiring hospital attendance. Baseline CGA data were obtained from routine clinical care and extracted from the registry for research purposes; the study investigators did not reassess participants after data extraction. All baseline registry assessments were performed by the gerontologist co-author using standardized YAŞAM assessment forms as part of routine care. Formal inter-rater reliability data were not available for this retrospective registry-based analysis.

### CGA domains and variable definitions

The baseline CGA domains included frailty, basic and instrumental function, cognition, mood, nutrition, and mobility/performance.

Frailty was recorded using the 9-point Clinical Frailty Scale (CFS), with higher scores indicating greater frailty [5]. Basic activities of daily living were assessed using the 6-item Katz Activities of Daily Living (ADL) scale (range, 0–6), with higher scores indicating better basic functional independence [12]. Instrumental activities were assessed using the 8-item Lawton Instrumental Activities of Daily Living (IADL) scale (range, 0–8), with higher scores indicating better instrumental function [13].

Cognition was assessed using the Mini-Mental State Examination (MMSE; range, 0–30) [14]. In routine YAŞAM practice, MMSE was documented using education-appropriate forms. For analysis, we derived a single MMSE value per participant by using the educated-form score when available and otherwise using the low-education form score. Mood was assessed using the 15-item short-form Geriatric Depression Scale (GDS-15; range, 0–15), with higher scores indicating more depressive symptoms [15]. Nutritional status was assessed using the Mini Nutritional Assessment–Short Form (MNA-SF; range, 0–14), recorded in the registry as MNA/MND, with higher scores indicating better nutritional status [16].

Mobility/performance was assessed using the Timed Up and Go (TUG) and the five-times sit-to-stand test [17, 18]. The YAŞAM assessment forms define conventional completion-time thresholds for impaired mobility/performance (> 13.5 s for TUG and > 12 s for five-times sit-to-stand). However, the analyzed registry export retained only the final binary status recorded in routine care rather than the underlying continuous completion times. These variables were therefore analyzed in their registry-native binary format without post hoc reconstruction from continuous values; indicator = 1 denoted performance within the routine clinical threshold (TUG ≤ 13.5 s; five-times sit-to-stand ≤ 12 s), whereas indicator = 0 denoted performance beyond the threshold.

### Study procedures and outcome measures

We queried the hospital information system for ED presentations occurring within 12 months after each participant’s t0. Cohort inclusion was not conditioned on having an ED visit during follow-up. We operationalized EMS utilization using the ED registration/arrival-mode field and aggregated these records at the patient level.

The primary outcome was at least one ED presentation to the study hospital arriving via an EMS ambulance within 12 months after t0 (yes/no). Repeat EMS utilization, defined as ≥ 2 EMS-transported ED presentations within 12 months after t0, was prespecified as a secondary outcome. We also summarized the total number of EMS-transported ED presentations during follow-up as a descriptive measure of utilization intensity.

Because death during follow-up could preclude subsequent EMS-transported ED presentation, we additionally performed a time-to-event sensitivity analysis for the primary outcome. For this analysis, participants were followed from the baseline YAŞAM assessment until the first EMS-transported ED presentation, death before any EMS event, or 12 months after baseline, whichever occurred first.

As a supplementary exploratory analysis, we summarized ICD-10-coded presenting complaints/reasons for presentation recorded for EMS-transported ED presentations. In the extracted dataset, up to the first 10 ICD-10-coded presenting complaint entries associated with EMS-transported ED presentations during follow-up were retained per participant. These fields reflected administrative coding of reasons for presentation in routine hospital records rather than adjudicated final ED discharge diagnoses. We categorized ICD-10 codes for descriptive reporting using a prespecified mapping scheme based on ICD-10 chapters and clinically meaningful groupings. Complaint/diagnosis categories were treated as descriptive exploratory variables only and were not used as adjustment variables in the primary association analyses.

This outcome definition was institution-specific: it captured only EMS-transported ED presentations to our hospital and did not capture EMS activations that did not result in transport, transports to other hospitals, or region-wide EMS use outside our institutional data systems.

### Data sources, linkage, and data management

We extracted baseline CGA variables from the YAŞAM registry and follow-up ED variables from the hospital information system. We linked datasets within the institutional environment using a pseudonymized unique patient identifier. Direct personal identifiers were removed from the analytic dataset, and working files were stored on access-controlled institutional systems. We constructed a patient-level analysis dataset containing one row per participant with baseline predictors, the prespecified follow-up window, death status (when available), and primary and secondary EMS outcomes.

### Statistical analysis

We summarized baseline characteristics by primary outcome status (EMS-transported ED presentation within 12 months: yes/no) using counts (%) for categorical variables and means (standard deviations) or medians (interquartile ranges) for continuous variables, as appropriate. We compared groups using chi-square tests or Fisher’s exact tests for categorical variables and independent-samples t-tests or Mann–Whitney U tests for continuous variables.

We evaluated associations between baseline geriatric measures and the primary outcome using logistic regression. To preserve the clinical interpretability of individual CGA domains and to reduce instability arising from conceptual and statistical overlap among functional, cognitive, nutritional, and mobility measures, we fitted separate age- and sex-adjusted logistic regression models for each CGA predictor as the primary domain-specific association analyses. These models were intended to describe associations between each routinely collected CGA measure and subsequent EMS-transported ED use, rather than to estimate mutually independent predictive effects. We reported odds ratios (ORs) with 95% confidence intervals (CIs).

To help readers interpret overlap across CGA domains, we additionally fitted a mutually adjusted complete-case logistic regression model including age, sex, frailty, Katz ADL, Lawton IADL, MMSE, GDS-15, MNA-SF, TUG, and five-times sit-to-stand. We reported this model as a complementary multivariable analysis to illustrate attenuation of effects when CGA domains were considered jointly, rather than as a final clinical prediction model. We also examined collinearity diagnostics using variance inflation factors. Age- and sex-adjustment was intended to account for basic confounding, not to resolve multicollinearity among CGA domains.

For repeat utilization (≥ 2 EMS-transported presentations), we performed an exploratory patient-level logistic regression analysis. To model utilization intensity (number of EMS-transported ED presentations within 12 months), we used a count regression approach and selected a negative binomial model when overdispersion was present; otherwise, we used a Poisson model with robust standard errors.

As a sensitivity analysis addressing unequal follow-up due to death, we fitted cause-specific Cox proportional hazards models for time to first EMS-transported ED presentation within 12 months. Separate age- and sex-adjusted models were fitted for each CGA measure, mirroring the primary logistic analyses. Participants were followed from baseline until first EMS-transported ED presentation, death before any EMS event, or 12 months, whichever occurred first. In these models, death before EMS use was treated as a competing event and participants were censored at the time of death. We reported hazard ratios (HRs) with 95% CIs. We did not use recurrent-event survival models because the time-to-event analysis was prespecified as a sensitivity analysis for time to first EMS-transported ED presentation, whereas recurrent utilization was addressed separately using repeat-use logistic regression and count models. We retained logistic regression as the primary analytic approach because the prespecified primary endpoint was a fixed 12-month binary outcome.

We quantified missingness for each variable. Cohort assembly exclusions should be distinguished from variable-level missingness within the final analytic cohort: 18 individuals were excluded because no baseline CGA had been performed, whereas regression models in the final cohort were conducted using model-specific complete-case analyses. We reviewed missingness descriptively at the variable level. Because missingness was confined to a small number of predictor variables, primarily MMSE, GDS-15, and MNA-SF, whereas the outcome and core adjustment variables were complete, and because the primary analyses were conducted as separate domain-specific association models rather than as a single prediction model, we considered model-specific complete-case analysis appropriate. We did not formally classify missingness as missing completely at random, missing at random, or missing not at random, because the retrospective registry-based design and limited auxiliary information did not permit a robust assessment of the underlying missingness mechanism. We report model-specific sample sizes for all analyses. We performed allowable-range checks for each scale variable before analysis. No implausible out-of-range entries were identified in the variables retained for the primary analyses. We used two-sided tests and set statistical significance at *P* < 0.05 while emphasizing effect sizes and precision. We performed all statistical analyses using Jamovi (version 2.3.28).

### Sample size

The final cohort included 587 eligible adults aged ≥ 80 years with a baseline YAŞAM CGA during the prespecified period. We did not perform an a priori sample-size calculation because the sample size was fixed by case availability in the registry.

## Results

### Participants and follow-up

The cohort comprised 587 community-dwelling adults aged ≥ 80 years who underwent a baseline comprehensive geriatric assessment in the YAŞAM registry between October 5, 2023 and December 10, 2024 and were linkable to the hospital ED records. The median age was 84 years (IQR, 82–88), and 335/587 (57.1%) were female. During follow-up, 51/587 participants (8.7%) died. Of these, 26 died before any EMS-transported ED presentation and therefore represented competing events for the primary outcome. Death during follow-up was more frequent among participants with EMS-transported ED use than among non-users (25/159 [15.7%] vs. 26/428 [6.1%], *P* < 0.001). Table 1 summarizes baseline characteristics by EMS-transported ED use.

**Table 1:** Baseline characteristics by EMS-transported ED use within 12 months

Characteristic	No EMS-transported ED use (*n* = 428)	≥ 1 EMS-transported ED use (*n* = 159)	*P* value
Age, years	84.0 (82.0–88.0)	85.0 (83.0–88.0)	0.067
Clinical Frailty score	6 (4–6)	6 (5–7)	< 0.001
Katz ADL	5 (4–6)	4 (2–6)	< 0.001
Lawton IADL	7 (3–8)	3 (1–7)	< 0.001
MMSE	24 (19–27)	21 (15.5–26)	0.011
GDS-15	3 (1–6)	5 (2–10)	< 0.001
MNA-SF	11 (9–12)	10 (7–11)	< 0.001
Female sex	243 (56.8)	92 (57.9)	0.887
Death during follow-up (EX = 1)	26 (6.1)	25 (15.7)	< 0.001
TUG indicator = 1	261 (61.0)	56 (35.2)	< 0.001
Five-times sit-to-stand indicator = 1	272 (63.6)	60 (37.7)	< 0.001

Values are median (IQR) or n (%). P values are from Mann–Whitney U tests (continuous/ordinal) or χ²/Fisher’s exact tests (categorical)

Abbreviations: ADL, activities of daily living; CI, confidence interval; GDS, Geriatric Depression Scale; IADL, instrumental activities of daily living; IQR, interquartile range; MMSE, Mini-Mental State Examination; MNA-SF, Mini Nutritional Assessment–Short Form; TUG, Timed Up & Go

### Primary outcome: any EMS-transported ED presentation within 12 months

Within 12 months of the index geriatric assessment, 159/587 participants (27.1%) had ≥ 1 ED presentation arriving via an EMS ambulance to the study hospital; 55/587 (9.4%) met criteria for repeat use (≥ 2 presentations), corresponding to 55/159 (34.6%) among users.

**Fig. 1:**
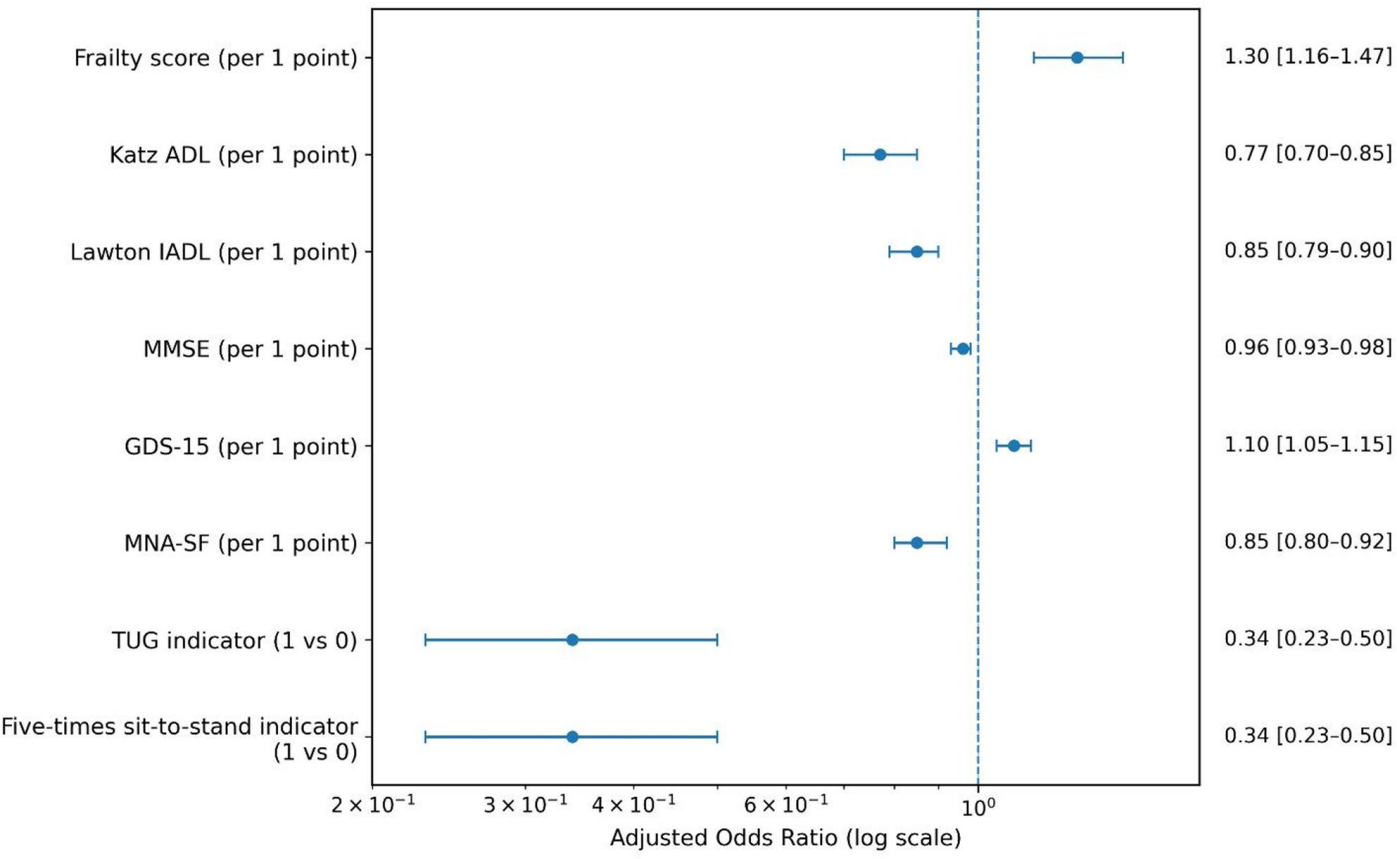
**Age- and sex-adjusted associations between baseline comprehensive geriatric assessment measures and EMS-transported emergency department use within 12 months.** Points indicate adjusted odds ratios (ORs), and horizontal bars indicate 95% confidence intervals (CIs) from separate logistic regression models adjusted for age and sex. The vertical dashed line denotes the null value (OR = 1.00). Higher values indicate higher odds of at least one EMS-transported ED presentation for frailty score and GDS-15, whereas higher values indicate lower odds for Katz ADL, Lawton IADL, MMSE, and MNA-SF. For binary mobility indicators, ORs compare indicator = 1 versus 0; indicator = 1 denotes performance within the routine clinical threshold (TUG ≤ 13.5 s; five-times sit-to-stand ≤ 12 s), whereas indicator = 0 denotes performance beyond the threshold, as recorded in the YAŞAM registry. Abbreviations: ADL, Activities of Daily Living; CI, confidence interval; ED, emergency department; GDS-15, 15-item Geriatric Depression Scale; IADL, Instrumental Activities of Daily Living; MMSE, Mini-Mental State Examination; MNA-SF, Mini Nutritional Assessment–Short Form; OR, odds ratio; TUG, Timed Up and Go; MMSE, Mini-Mental State Examination; MNA-SF, Mini Nutritional Assessment–Short Form; OR, odds ratio

Participants with EMS-transported ED use had higher frailty scores and lower functional scores than non-users (Table 1). Age- and sex-adjusted logistic regression models showed consistent associations between baseline geriatric measures and subsequent EMS-transported ED use (Table 2; Fig. 1). Higher frailty (OR per 1-point increase, 1.30; 95% CI, 1.16–1.47; *P* < 0.001) and higher depressive symptom scores (OR per 1-point increase in GDS-15, 1.10; 95% CI, 1.05–1.15; *P* < 0.001) were associated with higher odds of EMS-transported ED presentation. Although the association with frailty was statistically significant, the identical median CFS scores across groups and the substantial overlap in distributions suggest that this finding reflects gradations within an already highly frail population rather than separation between clearly distinct frailty categories. Higher Katz ADL, Lawton IADL, MMSE, and MNA-SF scores were associated with lower odds of EMS-transported ED presentation (Table 2). The binary mobility/performance indicators also showed some of the largest effect sizes among the separate age- and sex-adjusted domain-specific models (Table 2). AUC values are reported descriptively for these models to contextualize their discriminative performance; discrimination was modest overall (AUC range, 0.575–0.643), and Hosmer–Lemeshow tests did not indicate major departures from calibration (P range, 0.072–0.925) (Table 2, Panel A). Model sample sizes varied because MMSE was missing in 44/587 participants (7.5%), GDS-15 in 63/587 (10.7%), and MNA-SF in 4/587 (0.7%), whereas the other baseline CGA measures were complete. A summary of variable-level missingness is provided in Supplementary Table S1.

**Table 2:** Associations between baseline CGA measures and any EMS-transported ED use within 12 months

Baseline measure	*N*	Adjusted OR (95% CI)	*P* value	AUC	Hosmer–Lemeshow *P*
**Panel A. Separate age- and sex-adjusted domain-specific association analyses**					
Clinical Frailty score (per 1 point)	587	1.30 (1.16–1.47)	< 0.001	0.632	0.2692
Katz ADL (per 1 point)	587	0.77 (0.70–0.85)	< 0.001	0.639	0.6314
Lawton IADL (per 1 point)	587	0.85 (0.79–0.90)	< 0.001	0.640	0.5250
MMSE (per 1 point)	543	0.96 (0.93–0.98)	0.002	0.575	0.0720
GDS-15 (per 1 point)	524	1.10 (1.05–1.15)	< 0.001	0.622	0.6115
MNA-SF (per 1 point)	583	0.85 (0.80–0.92)	< 0.001	0.618	0.9252
TUG indicator (1 vs. 0)	587	0.34 (0.23–0.50)	< 0.001	0.643	0.9114
Five-times sit-to-stand indicator (1 vs. 0)	587	0.34 (0.23–0.50)	< 0.001	0.640	0.7131
**Panel B. Mutually adjusted complete-case logistic regression model**					
Variable	N	Adjusted OR (95% CI)	P value		
Age (per 1 year)	503	1.00 (0.96–1.05)	0.837		
Female sex	503	0.80 (0.51–1.26)	0.332		
Clinical Frailty score (per 1 point)	503	1.04 (0.86–1.25)	0.675		
Katz ADL (per 1 point)	503	0.88 (0.73–1.06)	0.180		
Lawton IADL (per 1 point)	503	1.01 (0.88–1.16)	0.865		
MMSE (per 1 point)	503	1.01 (0.97–1.05)	0.667		
GDS-15 (per 1 point)	503	1.02 (0.95–1.09)	0.620		
MNA-SF (per 1 point)	503	0.92 (0.83–1.02)	0.120		
TUG indicator (1 vs 0)	503	0.74 (0.33–1.64)	0.455		
Five-times sit-to-stand indicator (1 vs 0)	503	0.67 (0.32–1.43)	0.303		

Panel A shows separate logistic regression models adjusted for age and sex, presented as domain-specific association analyses rather than mutually independent predictive effects. AUC values are reported as descriptive discrimination metrics for these separate models and are not intended to imply stand-alone predictive utility. Panel B shows a complete-case mutually adjusted logistic regression model including age, sex, frailty, Katz ADL, Lawton IADL, MMSE, GDS-15, MNA-SF, TUG, and Five-times sit-to-stand. Variance inflation factors ranged from 1.09 to 3.77, suggesting moderate overlap among CGA domains without evidence of severe multicollinearity

Abbreviations: ADL, Activities of Daily Living; AUC, area under the receiver operating characteristic curve; CI, confidence interval; GDS-15, 15-item Geriatric Depression Scale; IADL, Instrumental Activities of Daily Living; MMSE, Mini-Mental State Examination; MNA-SF, Mini Nutritional Assessment short form; OR, odds ratio; TUG, Timed Up and Go

To improve clinical interpretability, we also examined a mutually adjusted complete-case model including age, sex, frailty, Katz ADL, Lawton IADL, MMSE, GDS-15, MNA-SF, TUG, and five times sit to stand (*n* = 503). In this model, effect estimates were substantially attenuated compared with the separate age- and sex-adjusted models, and no single CGA domain remained statistically significant (Table 2, Panel B). For example, frailty was associated with an OR of 1.04 (95% CI, 0.86–1.25), Katz ADL with an OR of 0.88 (95% CI, 0.73–1.06), Lawton IADL with an OR of 1.01 (95% CI, 0.88–1.16), MMSE with an OR of 1.01 (95% CI, 0.97–1.05), GDS-15 with an OR of 1.02 (95% CI, 0.95–1.09), MNA-SF with an OR of 0.92 (95% CI, 0.83–1.02), TUG indicator with an OR of 0.74 (95% CI, 0.33–1.64), and five times sit to stand indicator with an OR of 0.67 (95% CI, 0.32–1.43). Variance inflation factors ranged from 1.09 to 3.77, suggesting moderate overlap among CGA domains without evidence of severe multicollinearity.

In sensitivity analyses using cause-specific Cox proportional hazards models for time to first EMS-transported ED presentation, the direction and magnitude of associations were similar to those observed in the primary logistic models (Supplementary Table S2). Higher frailty remained associated with a higher hazard of EMS-transported ED use (HR 1.30; 95% CI, 1.17–1.45), whereas higher Katz ADL (HR 0.79; 95% CI, 0.73–0.85), Lawton IADL (HR 0.86; 95% CI, 0.81–0.90), MMSE (HR 0.96; 95% CI, 0.94–0.98), and MNA-SF scores (HR 0.87; 95% CI, 0.82–0.91) remained associated with lower hazard. Higher GDS-15 scores remained associated with increased hazard (HR 1.09; 95% CI, 1.05–1.13), and the mobility/performance indicators remained protective (TUG indicator: HR 0.37; 95% CI, 0.27–0.52; Five times sit to stand indicator: HR 0.37; 95% CI, 0.27–0.51).

### Secondary outcomes: repeat use and utilization intensity

Among all participants, repeat EMS-transported ED use (≥ 2 presentations) occurred in 55/587 (9.4%). In age- and sex-adjusted exploratory models, higher frailty and higher GDS-15 scores were associated with higher odds of repeat use, whereas higher Katz ADL, Lawton IADL, MMSE, and MNA-SF scores were associated with lower odds of repeat use; favorable mobility/performance indicator status was also associated with lower odds of repeat use (Table 3). The total number of EMS-transported ED presentations over 12 months ranged from 0 to 6; among users, the median was 1 (IQR, 1–2). In negative binomial models for the number of EMS-transported ED presentations, higher frailty and higher GDS-15 scores were associated with higher event rates, whereas higher functional, cognitive, and nutritional scores, as well as favorable mobility/performance indicator status, were associated with lower event rates (Table 3).

**Table 3:** Secondary outcomes: repeat use and utilization intensity (exploratory)

Baseline measure	*N*	Repeat use: adjusted OR (95% CI)	*P* value	*N*	Intensity: adjusted IRR (95% CI)	*P* value
Clinical Frailty score (per 1 point)	587	1.44 (1.18–1.76)	< 0.001	587	1.33 (1.20–1.48)	< 0.001
Katz ADL (per 1 point)	587	0.70 (0.61–0.80)	< 0.001	587	0.76 (0.70–0.82)	< 0.001
Lawton IADL (per 1 point)	587	0.80 (0.73–0.89)	< 0.001	587	0.84 (0.79–0.88)	< 0.001
MMSE (per 1 point)	543	0.94 (0.90–0.98)	0.005	543	0.96 (0.94–0.99)	0.006
GDS-15 (per 1 point)	524	1.15 (1.07–1.22)	< 0.001	524	1.09 (1.05–1.14)	< 0.001
MNA-SF (per 1 point)	583	0.79 (0.72–0.87)	< 0.001	583	0.84 (0.79–0.89)	< 0.001
TUG indicator (1 vs. 0)	587	0.22 (0.11–0.42)	< 0.001	587	0.32 (0.23–0.45)	< 0.001
Five-times sit-to-stand indicator (1 vs. 0)	587	0.17 (0.09–0.34)	< 0.001	587	0.31 (0.22–0.43)	< 0.001

Repeat use models are logistic regressions (≥ 2 vs. < 2 EMS-transported ED presentations) adjusted for age and sex. Intensity models are negative binomial regressions for the count of EMS-transported ED presentations (incidence rate ratio [IRR]) adjusted for age and sex. For binary mobility indicators, indicator = 1 denotes performance within the routine clinical threshold (TUG ≤ 13.5 s; five-times sit-to-stand ≤ 12 s), whereas indicator = 0 denotes performance beyond the threshold

Abbreviations: IRR, incidence rate ratio; OR, odds ratio

### Supplementary exploratory descriptive findings

A supplementary exploratory summary of administratively coded ICD-10 presenting complaints among EMS-transported ED presentations is provided in Supplementary Table S3. These codes were retained for descriptive context only and were not used as adjustment variables in the primary association analyses.

## Discussion

In this cohort of community-dwelling adults aged ≥ 80 years who underwent a standardized CGA, multiple routinely collected CGA domains were associated with subsequent EMS-transported emergency department presentations to the study hospital within 12 months. In separate age- and sex-adjusted models, higher CFS scores and greater depressive symptom burden, together with lower functional, cognitive, and nutritional scores, were associated with greater odds of EMS-transported ED use. Similar directional patterns were also observed for repeat use and transport intensity, suggesting that routinely collected CGA measures capture clinically meaningful vulnerability preceding emergency transport. Death during follow-up was a clinically important competing event in this population because it could preclude subsequent EMS-transported ED use. Although our primary analysis used a prespecified 12-month binary outcome, the direction and magnitude of associations were materially similar in cause-specific Cox sensitivity analyses for time to first EMS-transported ED presentation, supporting the robustness of the main findings. When these CGA domains were entered jointly into a mutually adjusted model, effect estimates were substantially attenuated and no single domain remained statistically significant, underscoring that these measures are better interpreted as overlapping markers of geriatric vulnerability than as independent stand-alone predictors. Among the separate age- and sex-adjusted domain-specific models, the mobility/performance indicators yielded some of the largest effect sizes, although these associations also attenuated substantially in the mutually adjusted complete-case model.

A first major finding was the central role of frailty in relation to EMS-transported ED utilization. In our analyses, higher CFS scores were associated with greater likelihood of any EMS transport and with repeat/intensive utilization patterns. This aligns with the growing evidence that frailty is highly prevalent among patients conveyed by ambulance and is tightly coupled to adverse trajectories, including complex care needs and higher service workload [1, 4]. In parallel, ED-based data from Europe demonstrate that frailty is common among older ED attendees, highlighting that emergency care systems frequently encounter frailty as a dominant background condition rather than a niche geriatric label [7]. Mechanistically, frailty reflects diminished physiological reserve and dysregulated homeostatic responses, such that relatively minor stressors (infection, dehydration, medication changes, a low-mechanism fall) can precipitate disproportionate functional and cardiopulmonary decompensation [6]. The CFS is designed to summarize this reserve across physical function and dependence, which plausibly explains why incremental frailty severity translated into higher reliance on emergency transport in our very old, community-dwelling population [5].

A second major finding was the strong and internally consistent association between functional and mobility-related vulnerability and EMS-transported ED use. Lower Katz ADL and Lawton IADL scores were associated with higher odds of transport, and the mobility/performance indicators also showed some of the largest effect sizes in the separate age- and sex-adjusted models. Clinically, this pattern is plausible: dependence in basic and instrumental activities, together with impaired mobility/performance, may reduce the feasibility of self-transport, increase fall-related and “can’t cope” presentations, and lower the threshold for ambulance-supported access to acute care. At the same time, these findings should be interpreted cautiously. In our dataset, the mobility variables were retained in routine binary registry format rather than as continuously recorded completion times, and their associations were substantially attenuated in the mutually adjusted complete-case model. This suggests that mobility/performance measures may function less as isolated independent predictors than as clinically visible markers of broader geriatric vulnerability across multiple CGA domains. Prior ED work nevertheless supports the clinical relevance of mobility assessment, including TUG performance, for identifying older adults at risk of adverse functional trajectories after emergency care encounters [19].

Third, cognitive impairment, depressive symptoms, and poor nutritional status emerged as additional, mutually reinforcing drivers of EMS-transported ED utilization. Lower MMSE scores and higher GDS-15 scores were each associated with increased likelihood of transport and with repeat/intensive use; similarly, lower MNA-SF scores were associated with higher utilization. These domains plausibly interact: cognitive impairment compromises adherence, symptom recognition, and timely outpatient care-seeking; depressive symptoms reduce motivation for self-management and physical activity and may amplify perceived symptom severity; malnutrition contributes to sarcopenia, immune dysfunction, and frailty progression, thereby increasing susceptibility to infection, delirium, and falls. We speculate that these geriatric syndromes may function as a networked risk state, with nutritional and affective deterioration accelerating functional decline and thereby increasing EMS dependence, rather than acting as isolated predictors. Importantly, ED-based frailty studies increasingly recognize that multidomain vulnerability predicts adverse outcomes beyond mortality, including return visits and readmissions, although effect sizes and performance vary by instrument and setting [7, 14–16, 20].

A fourth finding was that repeat and high-intensity EMS-transported ED use clustered within the already vulnerable subgroup, and the same CGA domains that were associated with any transport also showed coherent gradients for repeat use and counts. This aligns with broader EMS literature describing a relatively small subgroup of frequent users who contribute disproportionately to EMS workload and who often have complex multimorbidity and vulnerability profiles [3]. In our cohort, function and mobility-related vulnerability appeared particularly prominent within this pattern, supporting the potential value of proactive geriatric follow-up strategies targeted to the highest-risk subgroup.

From an implementation perspective, our findings may be more relevant to upstream models of care than to on-scene EMS decision-making itself. The observed associations suggest that routinely collected geriatric assessment domains may help identify very old adults with accumulating vulnerability before emergency transport becomes necessary. In this context, EMS-transported ED use should be interpreted primarily as an outcome of upstream frailty, functional dependence, and care gaps rather than as the preferred point of intervention. Accordingly, the practical implication of this study is not that single CGA-derived measures should be used as stand-alone tools in prehospital triage, but that they may help inform proactive case-finding, structured geriatric follow-up, home-based care pathways, or other community-based anticipatory services for high-risk older adults. This interpretation is also consistent with the modest discrimination of the individual domain-specific models and with the attenuation of effects in the mutually adjusted analysis. More broadly, it aligns with the service-planning rationale introduced earlier, namely that a relatively small subgroup of vulnerable older adults may contribute disproportionately to EMS demand and may therefore be particularly relevant for proactive, upstream models of care.

### Limitations

This study has several limitations. First, the design was observational and based on linkage to a single hospital’s ED, so we likely underestimated true EMS use if participants were conveyed elsewhere or if EMS attendances did not result in transport. We partially mitigated this by using a clear, objective primary outcome, arrival by EMS to the study ED, and a defined follow-up window, but some outcome misclassification remains possible. Because our outcome captured only EMS-transported ED presentations to the study hospital, the absolute rate of EMS utilization in this cohort is likely underestimated. In addition, if transport destination was associated with frailty, illness severity, geography, referral patterns, or other patient characteristics, differential misclassification may also have affected the observed associations, potentially biasing effect estimates in either direction. As we did not have access to a system-wide EMS database, we could not quantify the magnitude or direction of this potential bias. We also acknowledge that death may compete with the occurrence of EMS-transported ED presentation in very old adults. Although we addressed this issue in a cause-specific Cox sensitivity analysis, our primary models were logistic models based on a fixed 12-month endpoint and therefore do not fully capture the complexity of competing risks or recurrent-event processes. Second, our regression approach evaluated each CGA measure in separate age- and sex-adjusted models; therefore, residual confounding by comorbidity, social support, and access to primary care may persist, and the reported associations should not be interpreted as independent causal effects. Third, some baseline CGA measures were missing for a subset of participants, particularly MMSE and GDS-15, and the primary analyses therefore relied on model-specific complete-case analysis rather than multiple imputation. If missingness was related to unmeasured clinical or functional characteristics, some degree of selection bias may remain. Fourth, all baseline CGA assessments were performed by a single gerontologist as part of routine care. Although this may have supported procedural consistency, formal inter-rater reliability data were not available, and some measurement error cannot be excluded. Fifth, the mobility and performance variables were available only as registry-native binary indicators rather than continuous completion times, which limited measurement granularity and may have influenced the apparent strength of these associations. Sixth, because the cohort consisted of community-dwelling adults aged ≥ 80 years referred for CGA within an institutional YAŞAM registry linked to a single tertiary hospital in one region of Türkiye, the external validity of the findings is limited. The observed associations may partly reflect local care pathways, family support structures, referral practices, healthcare access, and EMS utilization thresholds specific to this setting. Therefore, caution is warranted when extrapolating these findings to broader older populations, including those living in other regions, rural areas, institutional care settings, or healthcare systems without comparable CGA-based follow-up structures. Finally, the study relied on a single baseline CGA assessment to examine EMS-transported ED use over the subsequent 12 months. Some geriatric domains, particularly frailty, mood, nutritional status, and functional capacity, may change over time, especially after intercurrent illness, ED presentation, or hospitalization. Accordingly, baseline CGA measures should be interpreted as time-specific markers of vulnerability rather than stable long-term determinants of EMS utilization.

Balanced against these constraints, the study also has notable strengths: a well-characterized oldest-old cohort with standardized baseline CGA across multiple geriatric domains; clinically relevant endpoints capturing both incident and repeat/intensity EMS-transported ED use; and internally consistent effect gradients across frailty, function, cognition, mood, nutrition, and performance measures, strengthening biological plausibility. The convergence of findings across domains supports the interpretation that CGA captures an underlying vulnerability state that meaningfully shapes prehospital emergency reliance.

## Conclusion

In community-dwelling adults aged ≥ 80 years, multiple routinely collected comprehensive geriatric assessment domains were associated with subsequent EMS-transported emergency department use within 12 months. Frailty, depressive symptoms, and lower functional, cognitive, nutritional, and mobility/performance status showed coherent associations with EMS-transported ED presentations, but these effects attenuated substantially when CGA domains were considered jointly. These findings suggest that routinely collected CGA measures are better interpreted as overlapping markers of geriatric vulnerability than as stand-alone predictive tools. Given the modest discrimination of the individual models and the institution-specific capture of EMS-transported ED presentations, these results should be interpreted cautiously and primarily as hypothesis-generating support for proactive, upstream models of care for high-risk older adults.

## Supplementary Information

Below is the link to the electronic supplementary material.

Supplementary Material 1

Supplementary Material 2

Supplementary Material 3

Supplementary Material 4

## Data Availability

The data underlying this article include individual-level health information derived from the YAŞAM comprehensive geriatric assessment registry linked to emergency department records. Due to privacy, data protection regulations, and institutional governance restrictions, the dataset is not publicly available. De-identified data may be made available by the corresponding author upon reasonable request, contingent on institutional approvals and execution of an appropriate data-sharing agreement.

## Abbreviations

ADL: Activities of Daily Living
CGA: Comprehensive geriatric assessment
CFS: Clinical Frailty Scale
CI: Confidence interval
ED: Emergency department
EMS: Emergency medical services
GDS-15: 15-item Geriatric Depression Scale
HR: Hazard ratio
IADL: Instrumental Activities of Daily Living
MMSE: Mini-Mental State Examination
MNA-SF: Mini Nutritional Assessment–Short Form
OR: Odds ratio
TUG: Timed Up and Go
