# Supplementary material for: Comprehensive geriatric assessment measures and subsequent EMS-transported emergency department use in adults aged ≥ 80 years: a retrospective cohort study
Source: BMC Emerg Med. 2026 Apr 18;26:157. doi: 10.1186/s12873-026-01590-z (PMC13224462; doi:10.1186/s12873-026-01590-z)
Supplement: Supplementary file 1 — Supplementary Material 1 [file 12873_2026_1590_MOESM1_ESM.docx]

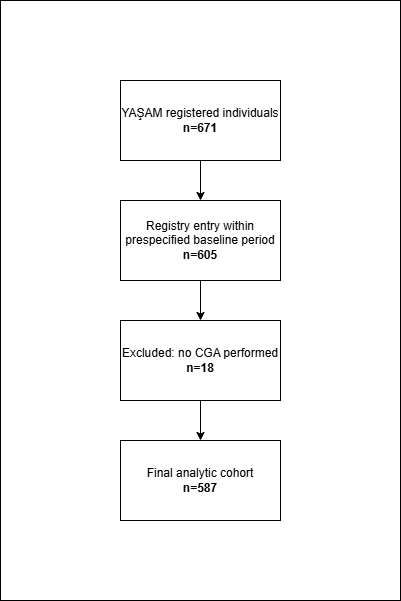


**Supplementary Figure S1. Cohort assembly from the institutional YAŞAM registry.**
Among 671 registered individuals, 605 had an eligible registry entry within the prespecified baseline period. Eighteen were excluded because no comprehensive geriatric assessment (CGA) had been performed, yielding a final analytic cohort of 587 participants.
