# Supplementary material for: Comprehensive geriatric assessment measures and subsequent EMS-transported emergency department use in adults aged ≥ 80 years: a retrospective cohort study
Source: BMC Emerg Med. 2026 Apr 18;26:157. doi: 10.1186/s12873-026-01590-z (PMC13224462; doi:10.1186/s12873-026-01590-z)
Supplement: Supplementary file 2 — Supplementary Material 2 [file 12873_2026_1590_MOESM2_ESM.docx]

| **Variable** | **Missing, n (%)** | **Available, n (%)** |
| --- | --- | --- |
| Age | 0 (0.0) | 587 (100.0) |
| Sex | 0 (0.0) | 587 (100.0) |
| Frailty score | 0 (0.0) | 587 (100.0) |
| Katz ADL | 0 (0.0) | 587 (100.0) |
| Lawton IADL | 0 (0.0) | 587 (100.0) |
| MMSE (derived combined score) | 44 (7.5) | 543 (92.5) |
| GDS-15 | 63 (10.7) | 524 (89.3) |
| MNA-SF | 4 (0.7) | 583 (99.3) |
| Timed Up and Go indicator | 0 (0.0) | 587 (100.0) |
| Five-times sit-to-stand indicator | 0 (0.0) | 587 (100.0) |
| Primary outcome (any EMS-transported ED use) | 0 (0.0) | 587 (100.0) |

**Supplementary Table S1. Missingness summary for baseline variables used in the primary analyses.** MMSE was derived by using the educated-form score when available and otherwise the low-education form score. Missingness percentages are calculated using the final analytic cohort (n=587).
