# Supplementary material for: Comprehensive geriatric assessment measures and subsequent EMS-transported emergency department use in adults aged ≥ 80 years: a retrospective cohort study
Source: BMC Emerg Med. 2026 Apr 18;26:157. doi: 10.1186/s12873-026-01590-z (PMC13224462; doi:10.1186/s12873-026-01590-z)
Supplement: Supplementary file 3 — Supplementary Material 3 [file 12873_2026_1590_MOESM3_ESM.docx]

| **CGA measure** | **N** | **EMS events** | **Adjusted HR (95% CI)** | **P value** |
| --- | --- | --- | --- | --- |
| Frailty score | 587 | 159 | 1.30 (1.17–1.45) | <0.001 |
| Katz ADL | 587 | 159 | 0.79 (0.73–0.85) | <0.001 |
| Lawton IADL | 587 | 159 | 0.86 (0.81–0.90) | <0.001 |
| MMSE | 543 | 143 | 0.96 (0.94–0.98) | <0.001 |
| GDS-15 | 524 | 138 | 1.09 (1.05–1.13) | <0.001 |
| MNA-SF | 583 | 158 | 0.87 (0.82–0.91) | <0.001 |
| Timed Up and Go indicator | 587 | 159 | 0.37 (0.27–0.52) | <0.001 |
| Timed Chair Stand indicator | 587 | 159 | 0.37 (0.27–0.51) | <0.001 |

**Supplementary Table S2. Sensitivity analysis using cause-specific Cox proportional hazards models for time to first EMS-transported emergency department presentation within 12 months.** Cause-specific Cox proportional hazards models were fitted for time to first EMS-transported emergency department presentation within 12 months after baseline. Separate models were constructed for each CGA measure, adjusting for age and sex. Participants were followed from baseline until first EMS-transported ED presentation, death before any EMS event, or 12 months, whichever occurred first. Death before EMS use was treated as a competing event and participants were censored at the time of death in the cause-specific analysis. During follow-up, 51 participants died; 26 of these deaths occurred before any EMS-transported ED presentation. Sample size varied across models because of missing data in MMSE, GDS-15, and MNA-SF.
