# Supplementary material for: Comprehensive geriatric assessment measures and subsequent EMS-transported emergency department use in adults aged ≥ 80 years: a retrospective cohort study
Source: BMC Emerg Med. 2026 Apr 18;26:157. doi: 10.1186/s12873-026-01590-z (PMC13224462; doi:10.1186/s12873-026-01590-z)
Supplement: Supplementary file 4 — Supplementary Material 4 [file 12873_2026_1590_MOESM4_ESM.docx]

**Panel A. Distribution by ICD-10 chapter/group**

| **ICD-10 chapter/group** | **n** | **%** |
| --- | --- | --- |
| Symptoms, signs, and abnormal clinical/laboratory findings, not elsewhere classified (R codes) | 188 | 72 |
| Injury/external causes (W and T codes) | 28 | 11 |
| Diseases of the digestive system (K codes) | 13 | 5 |
| Diseases of the musculoskeletal system and connective tissue (M codes) | 12 | 4.6 |
| Diseases of the genitourinary system (N codes) | 9 | 3.5 |
| Diseases of the circulatory system (I codes) | 5 | 1.9 |
| Diseases of the skin and subcutaneous tissue (L codes) | 3 | 1.2 |
| Mental and behavioural disorders (F codes) | 1 | 0.4 |
| Diseases of the respiratory system (J codes) | 1 | 0.4 |
| **Total** | **260** | **100** |

**Panel B. Most frequent individual ICD-10-coded presenting complaints**

| **ICD-10 code** | **Presenting complaint** | **n** | **%** |
| --- | --- | --- | --- |
| R06.0 | Dyspnea | 65 | 25 |
| W19 | Unspecified fall | 27 | 10.4 |
| R55 | Syncope and collapse | 22 | 8.5 |
| R53 | Malaise and fatigue | 17 | 6.5 |
| R07.4 | Chest pain, unspecified | 16 | 6.2 |
| R10.4 | Abdominal pain | 13 | 5 |
| R50.9 | Fever, unspecified | 10 | 3.8 |
| N39.0 | Urinary tract infection, site not specified | 9 | 3.5 |
| M79.1 | Myalgia | 7 | 2.7 |
| R51 | Headache | 7 | 2.7 |
| K92.2 | Gastrointestinal hemorrhage, unspecified | 6 | 2.3 |
| M54.5 | Low back pain | 5 | 1.9 |
| R11 | Nausea and vomiting | 5 | 1.9 |
| R30.0 | Dysuria | 5 | 1.9 |
| R07.0 | Pain in throat | 4 | 1.5 |
| R31 | Hematuria | 4 | 1.5 |
| R39.1 | Other difficulties with micturition | 4 | 1.5 |
| R42 | Dizziness and giddiness | 4 | 1.5 |

**Supplementary Table S3. Administratively coded ICD-10 presenting complaints among EMS-transported ED presentations during follow-up.** Counts refer to ICD-10-coded presenting complaint entries, not unique patients. The table includes 260 administratively coded entries recorded among 159 participants with at least one EMS-transported ED presentation during follow-up. Up to the first 10 ICD-10-coded presenting complaint fields per participant were retained from the hospital information system. These codes reflect routinely recorded presenting complaints/reasons for presentation rather than adjudicated final ED discharge diagnoses and were used for descriptive exploratory purposes only.
